# Donor and recipient contribution to phenotypic traits and the expression of biomineralisation genes in the pearl oyster model *Pinctada margaritifera*

**DOI:** 10.1038/s41598-017-02457-x

**Published:** 2017-06-02

**Authors:** Carole Blay, Serge Planes, Chin-Long KY

**Affiliations:** 1 Ifremer, UMR EIO241, Labex Corail, Centre du Pacifique, BP 49, Taravao, 98719 Tahiti French Polynesia; 2 PSL Research University, EPHE-UPVD-CNRS, USR 3278 CRIOBE, Université de Perpignan, 52 Avenue Paul Alduy, 66860 Perpignan, Cedex France; 3 Laboratoire d’Excellence “CORAIL”, 52 Avenue Paul Alduy, 66860 Perpignan, Cedex France

## Abstract

Grafting associates two distinct genotypes, each of which maintains its own genetic identity throughout the life of the grafted organism. Grafting technology is well documented in the plant kingdom, but much less so in animals. The pearl oyster, *Pinctada margaritifera*, produces valuable pearls as a result of the biomineralisation process of a mantle graft from a donor inserted together with a nucleus into the gonad of a recipient oyster. To explore the respective roles of donor and recipient in pearl formation, a uniform experimental graft was designed using donor and recipient oysters monitored for their growth traits. At the same time, phenotypic parameters corresponding to pearl size and quality traits were recorded. Phenotypic interaction analysis demonstrated: 1) a positive correlation between recipient shell biometric parameters and pearl size, 2) an individual donor effect on cultured pearl quality traits. Furthermore, the expressions of biomineralisation biomarkers encoding proteins in the aragonite or prismatic layer showed: 1) higher gene expression levels of aragonite-related genes in the large donor phenotype in the graft tissue, and 2) correlation of gene expression in the pearl sac tissue with pearl quality traits and recipient biometric parameters. These results emphasize that pearl size is mainly driven by the recipient and that pearl quality traits are mainly driven by the donor.

## Introduction

From a genetic point of view, grafting creates a chimera of a single individual made up of genetic material issued from two (or more) distinct genomes, with each genome maintaining its own distinct genetic identity throughout the life of the grafted organism. Typically, grafting associates two tissues from different individuals, the recipient and graft, which form vascular connections and survive as a genetic chimera due to a unique symbiotic relationship^1^. The partners in a graft are known as the scion and root in plants and the donor and recipient in pearl oysters. The use of grafting technology in both plant cultivation and pearl production provides a means of improving quality in these industries. Grafting is a well-established practice that has many horticultural and biological uses. The numerous applications of plant grafting include vegetative propagation, avoidance of juvenility, cultivar change, creation of unusual growth forms, repair, size control, biotic and abiotic stress resistance and physiological improvement^1^. Some studies have examined the contribution of the scion; for example, a scion of a red-flowering rose grafted on a white rose stock will continue to produce red roses^1^. Grafting is commonly employed in agronomy to indirectly manipulate scion phenotype^2^. As for the host organism, rootstocks are commonly used to propagate selected scions to improve fruit tree tolerance to environmental stress, and to control tree size^3, 4^. Roots anchor plants in the ground to provide water and nutrients from the soil and can serve as storage organs. The root system is a crucial component in coordinating response to a range of abiotic and biotic stressors^5, 6^. The influence of rootstocks on yield productivity is being increasingly recognized to be as important as grafted scions. In the plant system, the genetic characteristics of the root appear to drive vegetative growth and the overall growth of the grafted system depending on environmental conditions^7^.

In the pearl oyster grafting process (*Pinctada* sp.), similarities to the scion–root system were suggested by studies on the contributions of the recipient and donor to the expression of the overall phenotype (including cultured pearl quality traits). In *Pinctada maxima*, results indicated low donor-related heritability of pearl size^8^, and xenografts between *P. maxima* and *P. margaritifera* demonstrated that individual donors significantly influence the color and the surface complexion of the pearl^9^. In *P. margaritifera*, one study demonstrated the differential influence of individual donors with black or red outer shell phenotypes, combined with green or yellow inner shell phenotypes, on pearl darkness level, color categories and luster^10^. Another study examined the phenotypic correlation between recipient shell weight and size of pearls in *P. fucata*, revealing a limited positive relationship whereby recipient oysters with heavier shells produced bigger pearls^11^. These results indicate potential underlying genetic correlations between the recipient or donor growth and pearl size, whereby selection for faster growing oysters might further improve pearl size.

In order to understand the rootstock–scion interaction, some reviews have investigated physiological and biochemical aspects^12^, or hormonal signaling^13^. In pearl oysters, earlier studies have examined the influence of genetics, environmental factors and their interactions on pearl quantitative and qualitative traits^14^. A study in *P. maxima* indicated that characteristics of both donors and hosts were correlated with pearl traits^15^. In *P. margaritifera*, one such study demonstrated the influence of grow-out sites on cultured pearl quality traits over a broad geographic scale between archipelagos in the South Pacific^16^. Another analyzed environmental influence on pearl size parameters in relation to the recipient oyster biometric parameters^17^. Although it is clear from these studies that both donor and recipient oysters are involved in pearl formation in *P. margaritifera*, the relative contributions have not yet been clearly identified. Despite much study on pearl oysters, there remains a knowledge gap about donor size influence on pearl phenotype.

In the present study, the goal was to improve our understanding of the mechanisms driving the phenotypic variability arising from the recipient and the donor. The working hypothesis was that the recipient oyster confers quantitative traits to the pearl while the individual donor oyster drives qualitative traits of the pearl. In other words, everything related to growth and transfer of energy would be driven by the recipient oyster, and everything related to the quality of the nacrein deposit would be driven by the donor of the graft. Within this framework we defined four specific experiments to determine respective donor and recipient effects. (1) We sought to discern whether the donor or recipient was the main driver of pearl growth rate by comparing different recipient-donor phenotype combinations. (2) We evaluated drivers of the pearl phenotype by comparing effects of different recipient–donor phenotype combinations on nacre deposit quality. (3) We measured biomineralisation potential through a comparison of quantitative gene expression in the mantle graft (donor only effect) and the pearl sac (donor + recipient effect). For this approach, a representative panel of eight genes encoding proteins involved in aragonite biomineralisation (Pif-177, MSI60 and Perline), calcite biomineralisation (Aspein, Shematrin and Prismalin) or proteins implicated in both layers (Nacrein) was screened^18–20^. Finally (4), we followed the kinetics of the recipient–donor influence on pearl formation by monitoring pearl size and quality parameters over 12 months. This information provides basic knowledge to help us to understand the donor-recipient correlations and their relative contributions in pearl oyster grafts.

## Results

Overall, a total of 289 cultured pearls were harvested and analyzed. Donor pearl oysters were divided into two distinct groups according to their shell size (dorso-ventral measurement): small donor oysters, denoted “QL”, and large ones, denoted “TL”. The distribution of QL and TL oysters are shown in Table 1. Results are presented in four sections relating to 1) the influence of donor and 2) recipient shell size effect on cultured pearl size and cultured pearl quality, 3) biomineralisation capabilities and 4) pearl development kinetics.

**Table 1:** *Pinctada margaritifera* juvenile (N = 1535) shell diameter summary data. Values below 3 cm correspond to the QL phenotype group (n = 393) and values above 4 cm correspond to the TL phenotype group (n = 292).

	QL	Medium	TL
N	393	850	292
Mean	2.51	3.60	4.68
(±SD)	(±0.44)	(±0.32)	(±0.39)
Minimum	1.17	3.03	4.06
Maximum	3.01	4.04	5.82

### QL vs. TL and individual donor influence on pearl phenotype

At grafting time, the donor oyster width, height and thickness were recorded, showing average measurements of 48.6 ± 5.2 mm, 48.6 ± 5.2 mm and 12.7 ± 1.4 mm, respectively, for the QL donor group. The TL donor group was significantly larger for each parameter (*p* < 0.0001) with 79.8 ± 6.3 mm for shell height, 79.9 ± 6.9 mm for shell width and 21.0 ± 2 mm for shell thickness.

**Figure 1:**
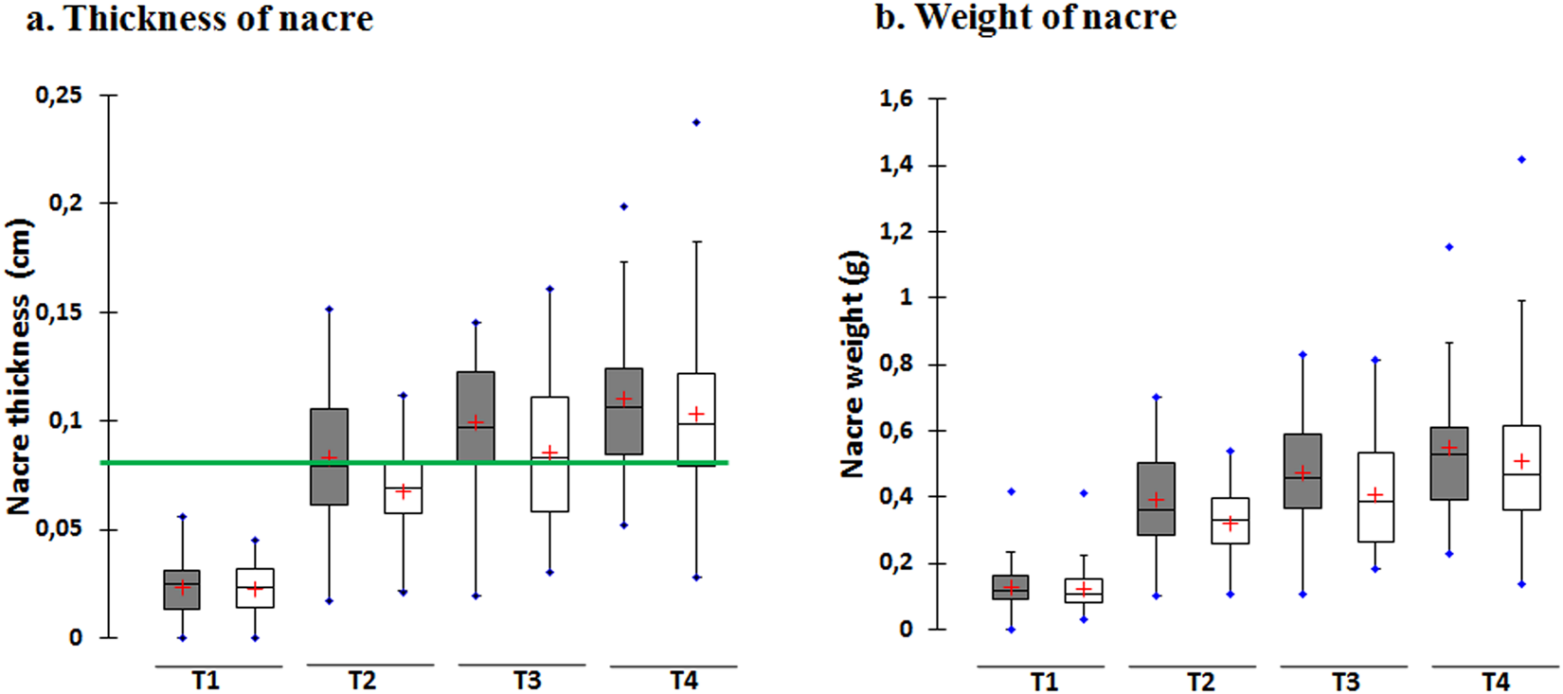
Box-plot showing: (**a**) thickness of nacre (cm) and (**b**). weight of nacre (**g**) on cultured pearls produced using graft tissue from each of the *P. margaritifera* donor phenotypes at each harvest time (T1 corresponds to 3 months of culture, T2 to 6 months, T3 to 9 months and T4 to 12 months). The box-plots in grey represent data for the QL donor phenotype and box-plots in white represent data for the TL donor phenotype. Each box-plot has the following 6 elements: 1) mean (“+” cross in the box-plot); 2) median (solid bar in the box-plot); 3) 25^th^ to 75^th^ percentile (rectangular box); 4) 1.5*interquartile range (non-outlier range of the box whiskers); 5) minimum and maximum values (extreme dots); and 6) outlier values (outside box whiskers). The green line on the nacre thickness box plot corresponds to the minimum nacre thickness necessary for pearl export outside French Polynesia (0.08 cm) (N = 289).

Comparing differences in the nacre produced between the QL and TL donor groups, we found that the average nacre weight and thickness of the QL donor group were 0.38 ± 0.22 g and 0.79 ± 0.04 mm, respectively (N = 142). In the TL donor group, the average nacre weight and thickness were 0.34 ± 0.20 g and 0.70 ± 0.04 mm (N = 147). No significant effect of donor oyster shell size phenotype was detected for either nacre weight or thickness (*p* = 0.058 and *p* = 0.065, respectively) (Fig. 1).

**Figure 2:**
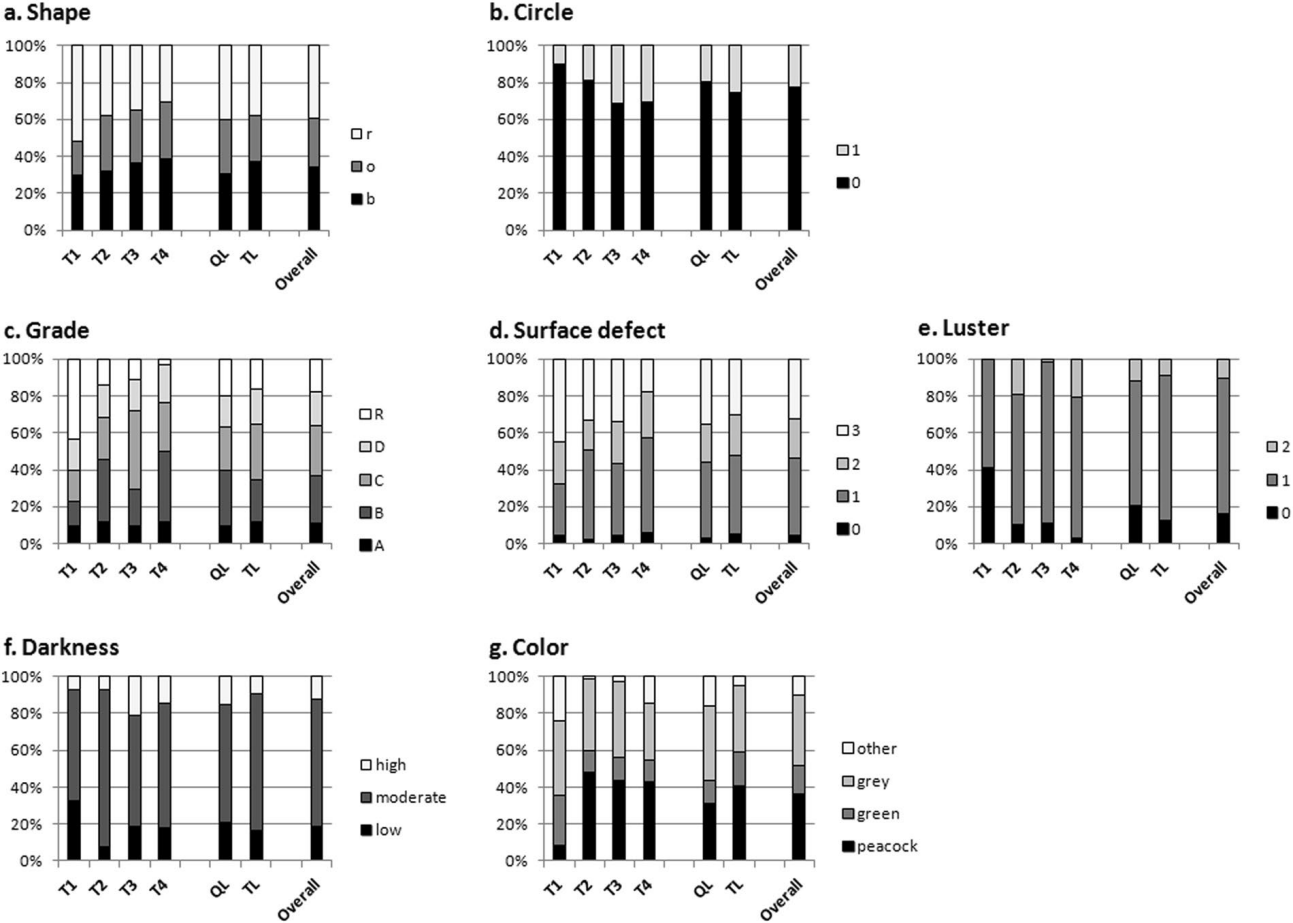
Cultured pearl quality traits from the experimental graft distribution. Percentage of cultured pearls for each harvest time (T1 corresponds to 3 months of culture, T2 to 6 months, T3 to 9 months and T4 to 12 months), for each donor phenotype (groups QL and TL) and for the overall harvest among the following variables are presented: (**a**) shape categories (“b” for baroque and semi baroque, “o” for oval and drop,” r” for round and semi round pearls), (**b**) pearl circles (“0” = absence and “1” = presence), (**c**) classification grade (“A” to “D” and Rejects), (**d**) surface defect classes (“0” = 0 defects, “1” = 1–5 defects, “2” = 6–10, and “3” = >10 defects), (**e**) luster levels (“0” = absence of luster, “1” = moderate luster, and “2” = high luster), (**f**) darkness level (low, moderate and high darkness) and (**g**) visual color categories (“peacock”, “green”, “grey” and “other”, corresponding to white and yellow pearls).

When effects of QL/TL donor phenotype were compared for subsequent pearl quality, however, no significant effect of donor oyster size group was observed for any pearl qualitative traits (darkness, luster, surface defects, grade, shape or presence of circle/s) except for color categories (Fig. 2), where the QL phenotype seems to give more pearls of “other” colors and less peacock pearls than the TL donor phenotype.

A significant individual donor effect was detected for nacre thickness (*p* = 0.045), but no significant individual donor effect was shown for nacre weight (*p* = 0.059). Pearls from donor “Q21” (N = 7 pearls) showed the greatest average nacre thickness (1.16 ± 0.50 mm) and nacre weight (0.566 ± 0.298 g) compared with donor “Q17”, which represented the minimum (N = 7 pearls) (0.45 ± 0.32 mm and 0.215 ± 0.141 g, respectively). The influence of donor size and the evolution of this relationship through time on qualitative pearl traits at harvest are illustrated in Figure 2.

A significant individual donor effect was detected for darkness level (*p* = 0.002), color category (*p* < 0.0001), cultured pearl luster (matte or shiny/glossy/highly glossy) (*p* = 0.0002) and the surface defect classes (*p* < 0.0001). However, no significant difference was recorded between the individual donors for the absence/presence of circles (*p* = 0.218) or for the three shape categories (*p* = 0.156).

### Recipient influence on pearl phenotype

**Figure 3:**
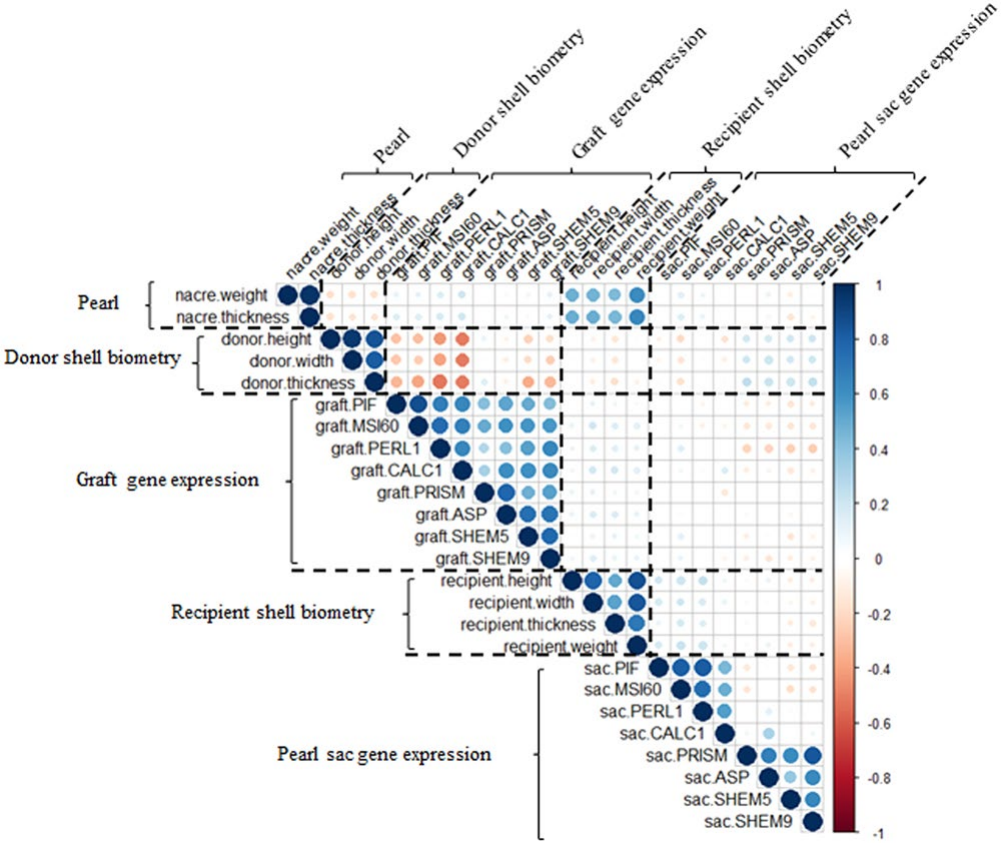
Correlation matrix, made using corrplot, between *Pinctada margaritifera* donor and recipient shell biometric parameters (shell height, width, and thickness, and total oyster weight (soft tissue parts + shells)), pearl size (nacre weight and thickness), and the relative expression of the 8 biomineralisation genes in the mantle graft and pearl sac. The areas of circles show the value of corresponding Spearman correlation coefficients. Correlation values are presented in the upper panel in circle; positive correlations are displayed in blue and negative correlations in red. Color intensity (light to dark) and the size of the circle (small to big) are proportional to the correlation coefficients (0 to 1 for the positive coefficient and 0 to −1 for negative coefficient) where for example the correlation coefficients on the principal diagonal is equal to 1 (it was represented in dark blue and biggest size of circle). The legend on the right side of the correlogram shows the Spearman correlation coefficients with their corresponding colors.

Nacre thickness and weight were both significantly correlated with recipient oyster shell biometric parameters (weight, height, thickness and width), Spearman rho coefficients ranged from 0.51 to 0.65 and 0.49 to 0.62, respectively. The highest significant positive correlations were found for nacre thickness and pearl weight with recipient pearl oyster weight (Rho = 0.65 and Rho = 0.62, respectively, *p* < 0.0001). In addition, there were significant positive correlations of nacre thickness and weight with shell width (+0.51 and +0.49, respectively, *p* < 0.0001) (Fig. 3).

### Molecular phenotype

**Figure 4:**
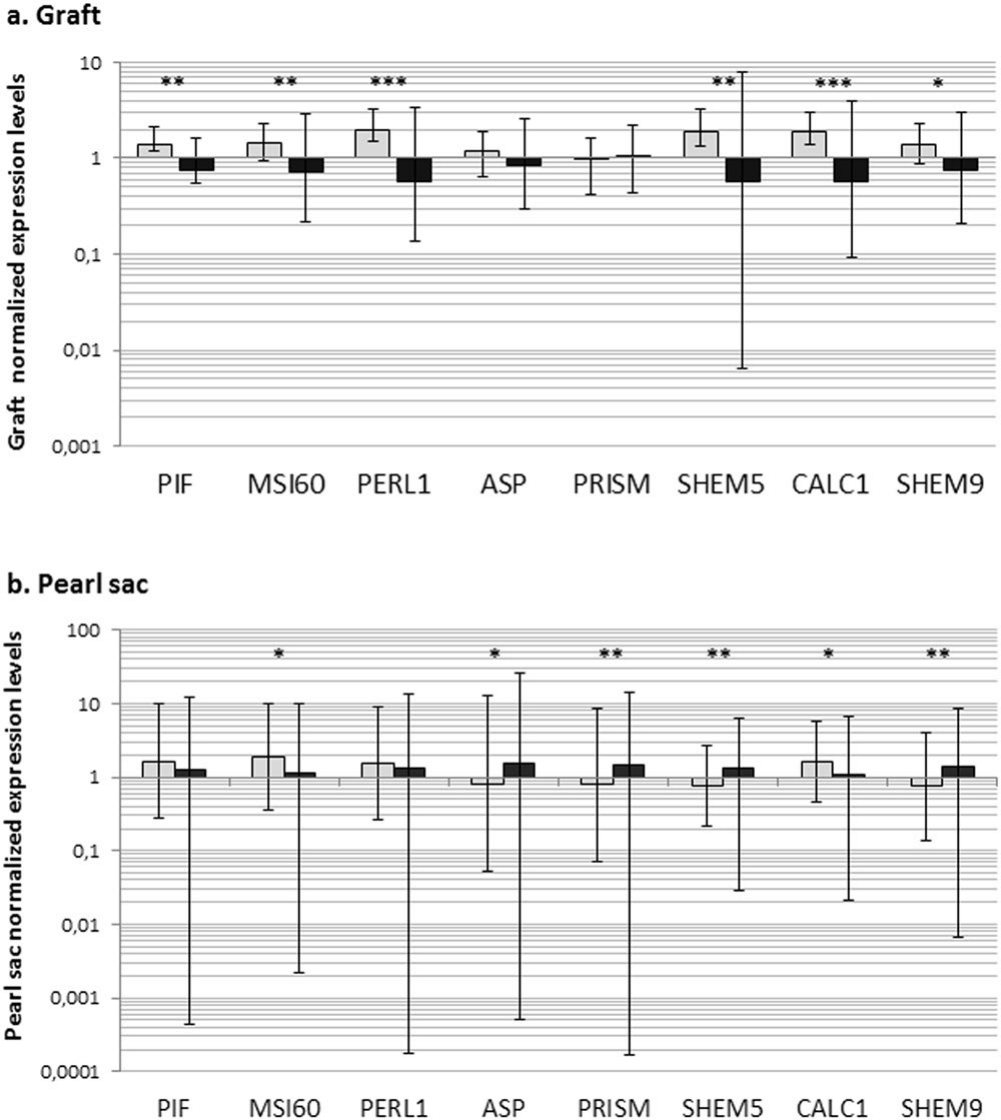
Relative expression of 8 biomineralisation genes in *P. margaritifera* in: (**a**) the mantle graft (N = 40) and (**b**) the pearl sac (N = 217 with 108 QL and 109 TL). Histograms in light grey represent data for the TL donor phenotype and histograms in dark grey represent data for the QL donor phenotype. Y axes are in logarithmic scale. Error bars correspond to standard deviations. Statistical differences between the two phenotypes are indicated by asterisks: * for 0.05 < *p* < 0.005, ** for 0.005 < *p* < 0.0005, *** for *p* < 0.0005”.

The relative expression levels of the eight biomineralisation genes analyzed in the donor oyster mantle graft tissue and pearl sacs from the QL and TL groups are shown in Fig. 4a. The comparison between the two groups of donors for the three aragonite-related genes, *Pif-177*, *MSI60 and Perl1*, showed significant higher gene expression levels in the TL group (*p* < 0.0001) in the graft tissue compared with the QL group. *Shem5*, *Shem 9* and *Calc1* also showed significant higher gene expression levels in the TL group (*p* < 0.0001) compared with the QL group.

All biometric parameters of the donor oysters were significantly correlated with the relative expression level of seven biomineralisation genes in the graft (*p* < 0.0001); only *Prism* was not correlated. Nacre thickness and pearl weight were not correlated with the relative expression level in the graft of eight biomineralisation genes (Fig. 3). Furthermore, the recipients shell height was correlated with the relative expression level of *Perl1* in the pearl sac (Rho = 0.236, *p* = 0.0004 for shell height) (Fig. 3).

Among the eight candidate genes studied in the pearl sac at the three harvest times, the expressions of six were significantly affected by donor phenotype (Fig. 4b). *MSI60* and *Calc1* gene expression levels were significantly higher for “TL” phenotypes compared with “QL” phenotypes (*p* = 0.009 and *p* = 0.014 respectively). *Prism*, *Asp*, *Shem5* and *Shem9* showed significantly greater expression in “QL” phenotypes compared with “TL” ones (*p* = 0.043, *p* = 0.049, *p* = 0.004 and *p* = 0.009, respectively).

No significant differences in relative expression of the panel of genes in the graft were found for pearl quality traits. In the pearl sac, however, significant effects were observed for the majority of the panel of genes, depending on grade, level of surface defects, luster and color (Supplementary Table S1).

### Pearl development kinetics

**Figure 5:**
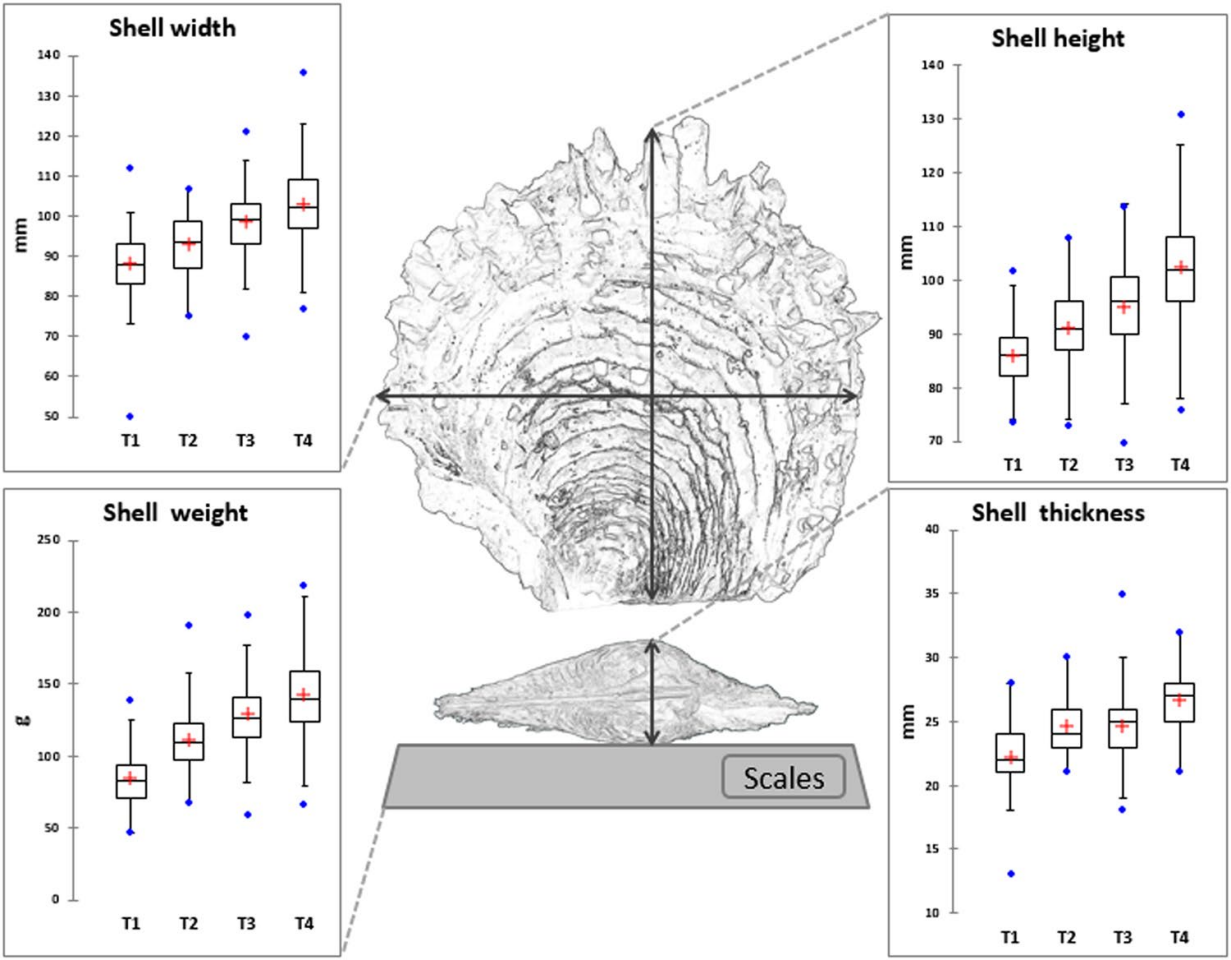
*Pinctada margaritifera* recipient oyster shell biometric parameters measured at each harvest time (T1 corresponds to 3 months of culture, T2 to 6 months, T3 to 9 months and T4 to 12 months of rearing): shell width, height, thickness, and the total oyster weight (soft tissue parts + shells). Each box-plot has the following 6 elements: 1) mean (“+” cross in the box-plot); 2) median (solid bar in the box-plot); 3) 25^th^ to 75^th^ percentile (rectangular box); 4) 1.5*interquartile range (non-outlier range of the box whiskers); 5) minimum and maximum values (extreme dots); and 6) outlier values (outside box whiskers) (N = 289).

Analyzing kinetics data over the 12 months of the study, we observed, as expected, a very highly significant difference in nacre thickness between harvest times (*p* < 0.0001). Mean nacre thicknesses at harvest times T1, T2, T3 and T4 were 0.02 ± 0.01, 0.08 ± 0.03, 0.09 ± 0.03 and 0.11 ± 0.03 cm, respectively. Nacre was significantly thinner at T1 than at T2, T3 and T4. T3 and T4 were not significantly different from one another. Pearl nacre weight was also highly significantly different between harvest times (*p* < 0.0001). Mean pearl weights at harvest times T1, T2, T3 and T4 were 0.12 ± 0.07, 0.35 ± 0.14, 0.44 ± 0.17 and 0.54 ± 0.20 g, respectively. T1 pearls were significantly lighter in weight than T2, T3 and T4 ones. As with nacre thickness, T3 and T4 were not significantly different from one another (Fig. 1). Measurements on recipient oysters showed highly significant differences between the four harvest times (*p* < *0.0001*), as shown in Fig. 5. Mean height (dorso-ventral measurement ± SE) for recipient shell at T1, T2, T3 and T4 harvest times were 86.38 ± 5.79, 91.42 ± 7.38, 96.15 ± 7.27 and 102.07 ± 7.61 mm, respectively. Mean weight for recipient shell at T1, T2, T3 and T4 harvest times were 84.99 ± 19.34, 110.38 ± 21.61, 130.87 ± 24.75 and 144.57 ± 26.14 g, respectively.

Comparison between harvest times showed no significant difference between the proportions of the three shape categories (*p* = 0.215). After 3 months of culture, the pearl shapes were distributed as follows: Baroque 29.6%, N = 21; Round 52%, N = 37; Oval 18%, N = 13. After 12 months of culture, the distribution had remained similar: Baroque 38%, N = 26; Round 31%, N = 21; Oval 31%, N = 21.

Figure 2  shows the ratios of pearls with and without circles at each harvest time. A highly significant effect was recorded for the presence or absence of circles (*p* = 0.005) between each of the harvest times. The T3 and T4 harvest times yielded about three times more circled pearls than T1.

Data analysis showed a highly significant difference in pearl luster and grade between harvest times (*p* < 0.0001). T1 pearls were significantly more “matte” and less “glossy” than T2, T3 and T4 pearls. T1 had significantly less grade B pearls (12.7%) and more Reject pearls (43.7%) than the other harvest times T2 (34.2% and 13.9%, respectively), T3 (19.7% and 11.3%) and T4 (38.2% and 2.9%).

A highly significant difference in the darkness of pearls was observed between the different harvest times (*p* = 0.0004). T1 harvest time had significantly more lighter-toned pearls (32.4%) than the later harvest times T2 (7.6%), T3 (18.3%) and T4 (12.7%). T3 and T4 had significantly more dark pearls (21.1% and 17.7%, respectively) than T1 (7%) and T2 (7.6%) (Figure 2).

For cultured pearl surface defects, no significant harvest time effect was detected (*p* = 0.067) (Figure 2).

## Discussion

Our approach, based on studying the influence of donor and recipient phenotypes on resulting pearl and molecular phenotypes, has yielded important results for pearl oyster breeding strategy. Overall, the results show that it is mainly the recipients that influence the size of the pearl, while the individual donors influence pearl quality traits (except for pearl shape and the presence/absence of circles, which seem to be more influenced by graft surgical procedure).

In the present study, we showed that pearl size phenotype (nacre weight and thickness) was highly correlated with the biometric parameters of the recipient oyster, while those of the donor oyster (QL/TL groupings) had no impact. This positive correlation confirms results observed in *P. fucata* ^11^ and in *P. maxima* ^15^. Furthermore we observe a significant positive correlation between the expression of the aragonite gene in the pearl sac (*Perl1*) and one recipient oyster biometric parameter (shell height). This result suggests that the recipient oyster affects the activities of the biomineralising tissues, which are mainly dependent on calcium metabolism in the epithelial tissue of the pearl sac^21^. The recipient oyster regulates the metabolism of the pearl sac by supplying nutrients during the period of culture and thus regulates the expression of the biomineralisation genes, especially those implicated in the aragonite layers^22^. High biomineralisation capabilities may have contributed to a greater nacre deposition, as already observed in *P. maxima* ^23–25^ and *P. fucata* ^26^. The recipient oyster can be compared to the rootstock of a grafted plant, with recipient oyster and rootstock each corresponding to a grafted organism. In the plant system, rootstocks are the driving component of production systems and studies have reported that they can influence tree growth, yield and fruit size, weight, and rind thickness^27–33^. However, it is not fully understood how the rootstock controls scion growth. The grafted organism seems to have a key role in growth owing to its nutritive function. Interestingly, the same principle applies in the animal system we studied. The positive relationship between pearl size (nacre weight and thickness) and weight of the recipient is valuable information because it can serve as a basis for selective breeding strategy for fast growing recipient pearl oysters. For mollusk species, between 10% and 20% improvement in selected growth traits can be obtained per generation^34, 35^.

A strong individual donor effect was observed on pearl quality traits, such as color (darkness and visual color categories) and quality parameters (luster, surface defects and grade). Pearl quality traits are influenced by a number of highly variable factors, including genetics, the environment and their interaction, as well as surgical technique and graft hygiene^36^. A recent study in *P. maxima* found significant individual donor effects for color, nacre deposition, luster, shape and defects; however, each of these traits was grouped into broad categories in this previous study, making specific comparisons between the two studies difficult^15^. In plant models, research on rootstock–scion interaction demonstrated that yield and quality are conferred by the scion cultivar. Scion quality clearly affects final yield and fruit quality in grafted plants, but rootstock effects can also impact these characteristics^4, 37^.

Expression analysis in the pearl oyster graft, based on a panel of genes encoding proteins implicated in the shell biomineralisation process, indicated significant differences of gene expression level between the QL and TL donor groups for the three genes of the panel implicated in aragonite formation (*Pif-177, MSI60*, and *Perl1*). The protein genes *Pif-177* and *MSI60* regulate growth, nucleation and the organization of the aragonite crystal^20, 22, 38^. Perline is the protein equivalent of the N14 protein identified in *P. maxima* ^39^ and seems to be specifically involved in the formation of the nacreous layer and promotion of aragonite crystal nucleation^23^. Aragonite-related gene expression levels were significantly higher in the graft tissue from the TL group than from the QL group. Some other genes (*Shematrin 5 and 9* and *Nacrein*) showed significantly higher expression levels in the graft originating from the TL group. *Shematrin 5 and 9* are implicated in the calcite mineralisation process, in which they facilitate the formation of calcite prisms^40^. These results seem to show the opposite trend to the pearl phenotype observations, since the TL and QL groups did not differ in nacre weight and thickness. The significantly higher expression levels of some genes in the TL group correspond to the biometric parameters of the donor oyster but not to the quantitative traits of the pearl. A study in *P. fucata* examined the differences of gene expression in large and small oysters. RNA-seq analysis revealed that the number of genes up-regulated in small oysters was greater compared to large oysters and could be explained by catch-up growth^41^. In the pearl sac analyses, we observed a relationship between relative gene expression in the pearl sac and grade, surface defects, luster and pearl color. The role of the pearl sac in nacreous layer biomineralisation is thought to mirror that of the oyster mantle. The gene-expression patterns of shell matrix proteins in pearl sacs were measured relative to the biomineralisation process of the nacreous layer^42^. The genome of the individual donor oyster and its influence on the pearl biomineralisation process remain active in the pearl sac tissue during the entire pearl culture period right up until harvest time^9, 43–45^, confirming the influence of individual donors on quality pearl traits.

Our examination of pearl formation over time (12 months of rearing) clearly showed that nacreous deposition is not linear. By the half-way point of the experiment (6 months), 69.7% of the final nacre thickness and 65% of the nacre weight had already been reached. At this point, nacre deposition slowed down. No previous study has examined the deposition of the nacreous layer on the pearl over culture time. However, some studies did examine the beginning stage (35 days) of nacreous layer deposition during pearl formation in *P*. *fucata* ^42, 46^ and in *P*. *margaritifera* ^47^. These found that the first CaCO_3_ deposited on the nucleus was the aragonitic^47^ or calcitic^46^ prismatic layer found in the cross section of the pearl, and that the nacreous layer then formed on top of this prismatic layer. The non-linear nacreous deposit seems to be influenced by the growth of the recipient oyster (whose biometric parameters are correlated with nacre weight and thickness). Shell growth is influenced by two major factors: microalgal concentration and temperature, both of which regulate the expression of most of the shell matrix protein genes implicated in the biomineralisation process^22^. However, recipient growth had a linear pattern over the 12 months of culture. Thus, the growth of the recipient seems to be an important driving factor of pearl growth but not the only influence implicated. The high influence of the recipient and the weak influence of the donor on pearl size at harvest suggest that their respective roles are not totally revealed here.

In our experiment, we also recorded that luster, grade and darkness of pearls improved with duration of culture, but we also noted an increase in the proportion of circled pearls. According to Wada^48^, who focused on surface color and pigmentation, the quality of nacreous pearls is determined by the ratio of the thickness of the lower prismatic layer to that of the upper nacreous layer. It was therefore logical that we found an improvement of quality grade over time, because nacre deposition increased with time of culture^49^. Individual donor oysters influence quality traits such as color, luster, amount of surface defects and grade but do not affect shape or the presence of circles. The differences observed between quality traits underline the complexity of their genetic basis and that of the donor/recipient interaction.

The present study is the first to identify the relative impact of donor/recipient oyster size on pearl size and quality traits during culture time in the marine bivalve *P. margaritifera*. It was demonstrated that 1) pearl size characters (nacre weight and thickness) are influenced by the recipient oyster phenotype and 2) the major qualitative traits (color, darkness, luster, and incidence of surface defects) are influenced by individual donor oysters. The recipient oyster shell parameters are correlated with pearl size, with the largest recipient giving the largest pearls. Similarly to other examples in the living world, the recipient organism seems to have a more important role than expected. Donor/recipient combinations should therefore be selected carefully. For the pearl industry, the contribution of the recipient found here suggests it would be beneficial to develop breeding programs on growth of recipient oysters in order to obtain larger and thus more valuable pearls. In parallel, a breeding program could be developed for quality and color of donor oysters in order to obtain high quality pearls in desired colors. Furthermore breeding program could be based on pedigree. In fact, genealogy information is essential for genetic traceability. The importance of maintaining pedigree records could aim to increase effective population sizes and minimise inbreeding to ensure long-term genetic gain, viability of aquaculture breeding programs and productivity by the development for example of a set of multiplex PCRs as in *C. gigas* oyster^50, 51^ or in the abalone *Haliotis midae* ^52^. The present work could serve as a basis for future studies exploring the donor/recipient relationship in greater depth.

## Materials and Methods

### Animals

**Figure 6:**
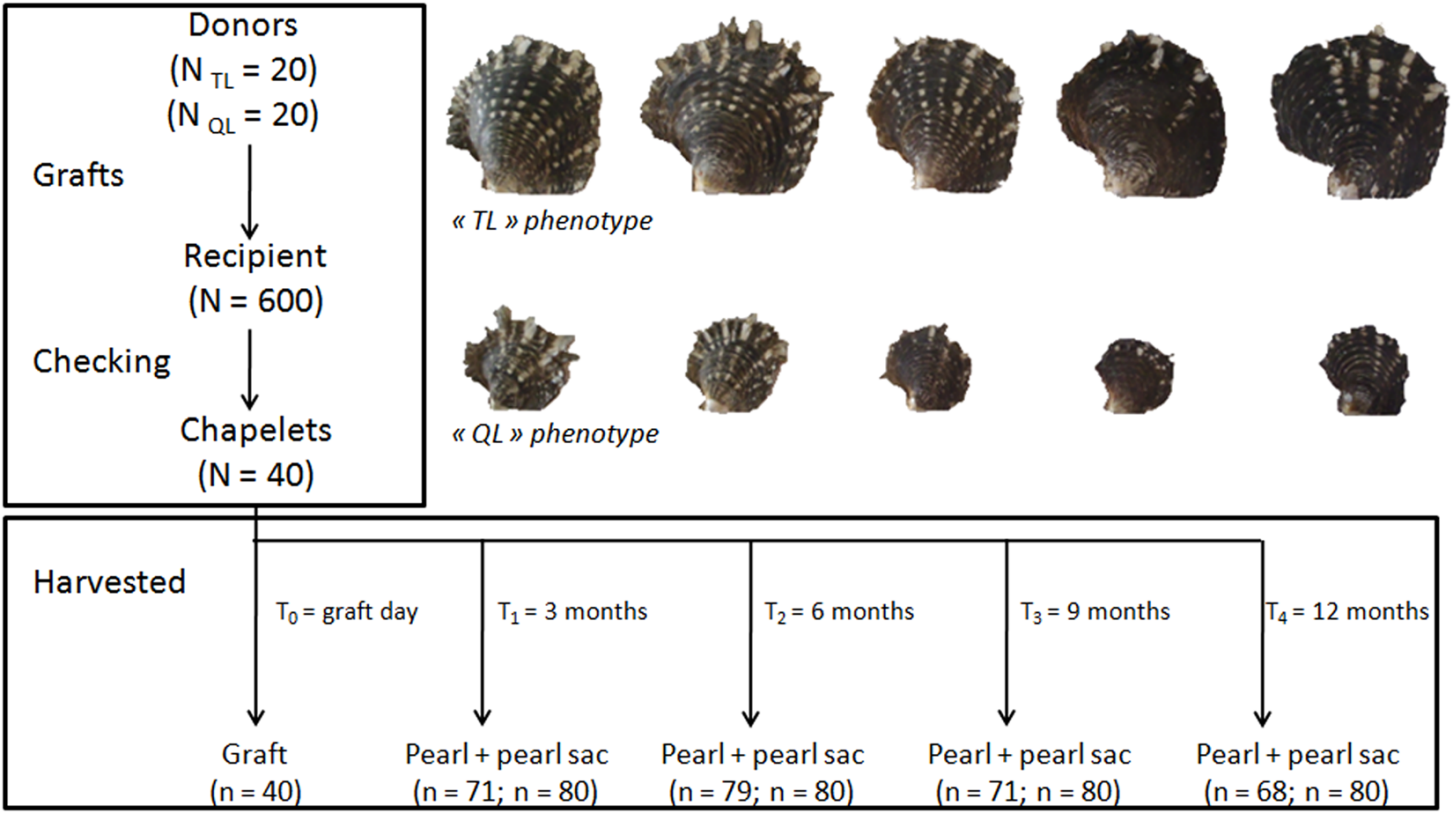
Experimental graft design for *P. margaritifera* donors using QL and TL phenotypes as donors. The grafts (N = 600) were performed on Regahiga Pearl farm (Mangareva Island, Gambier archipelago) then nucleus rejection and receiver mortality were evaluated 45 days after the graft operation (checking). Two recipient oysters per donor were randomly harvested every three months of the experiment.

Wild *Pinctada margaritifera* were collected as spat using commercial collectors in the lagoon of Takaroa atoll (Tuamutu archipelago, French Polynesia) in March 2013. Nine months later (December 2013), 20 of these collectors were transferred to Ifremer facilities in Vairao on Tahiti Island (Society Archipelago, French Polynesia). Pearl oysters (N = 1535) were then removed from the collectors and divided into two distinct groups according to their shell size (dorso-ventral measurement): small “QL”, (N = 393; 2.5 cm in means) and large “TL” (N = 292; 4.7 cm in means). The distribution and phenotypes of QL and TL oysters are shown in Table 1 and Fig. 6. These oysters were then tied onto a CTN (Cord Technical Nakasai) rearing system^53^, where they were left to grow, protected using plastic mesh to prevent predation, until they reached a sufficient size to start the grafting experiment. These oysters were then transferred to Regahiga Pearl Farm (Mangareva island, Gambier archipelago, French Polynesia) to be used as donors. Prior to nucleus implantation and graft surgery, oysters from the QL and TL groups were collected from the rearing station, detached, washed with a high pressure spray (to remove epibiota) and stored until the grafting operation.

### Grafting procedure

All grafts were performed under standard production conditions by a single expert at the Regahiga Pearl Farm so as to minimize the grafter effect on pearl quality traits described in ref. 54. The recipient oysters were issued from local natural spat collection in the Mangareva lagoon. A total of 40 donors (20 of the TL phenotype and 20 of the QL phenotype) were used to perform 600 grafts (20 grafts per donor for TL and 10 grafts per donor for QL) over a 2-day period, using 1.8 BU nuclei (5.45 mm diameter; Imai Seikaku Co. Ltd., Japan) (Fig. 6). During the grafting process, 3 to 5 graft tissue pieces of each donor oyster were sampled, preserved in RNAlater® and stored at −80 °C for subsequent RNA extraction. Recipient oysters were examined 45 days post grafting to estimate nucleus retention and mortality rates, as described in ref. 55.

### Measurements of shell biometric parameters and pearl growth rate

Prior to the grafting operation, shell height, width and thickness of the 40 donor oysters were measured using Vernier calipers. All recipient oysters were washed with a high pressure spray to remove epibionts and the following biometric measurements were taken: shell height, width, thickness, and total weight (soft tissue parts + shells) (to 0.01 g) (Fig. 5)^17^.

Once harvested, cultured pearls were cleaned by ultrasonication in soapy water with a LEO 801 laboratory cleaner (2-L capacity, 80 W, 46 kHz); they were then rinsed in distilled water. Some *keshi* (small non-nucleated nacre deposits) were also harvested but these were not graded in the present study. The size of the cultured pearls was assessed by measuring nacre thickness and weight^49^.

### Cultured pearl quality parameter measurement

Surface defects, luster and grade category of the cultured pearls were evaluated. Cultured pearl shape was characterized in two ways: the presence/absence of circle/s and the shape category (“b” for baroque and semi baroque, “o” for oval and drop, “r” for round and semi round pearls). Two kinds of color evaluation were made on the cultured pearls: the darkness of color and the visually-perceived color category (peacock, green, grey, other). Cultured pearl grade was determined for each sample according to the official Tahitian classification (Journal Officiel 2001 n° 30, 26 July 2001) from the most to least valuable quality: A, B, C, D and Rejects (*rebuts*). Briefly, the five grades are mostly based on surface purity and luster, from A (cultured pearls showing no surface defects or small defects confined to less than 10% of their surface and having very good luster;) to D (cultured pearls showing many highly visible defects over more than two thirds of their surface and having poor luster) and Rejects (cultured pearls that have too many defects to be graded). The latter are consequently discarded and ultimately destroyed. Finally, surface defects and luster (components of cultured pearl grade) were determined separately so that they could be studied independently. Quality traits were evaluated as described in ref. 56. To ensure homogeneity in parameter assessment, all evaluations were made visually (without a jeweler’s loupe) by the same operators.

### Biomineralisation gene expression analysis

Oyster shell and cultured pearls are formed by biomineralisation activities in two distinct tissues: the mantle of the recipient and the pearl sac in the gonad, formed from mantle tissue from the donor^57^. Around the nucleus, a pearl sac is formed by proliferation of the outer mantle epithelial cells of the mantle graft which secretes successive nacre layers on the nucleus^58, 59^. Pearl sac consists of mucous cells containing large acidophilic granules and epidermal cells^42^ that secrete proteins resulting into culture pearl formation, a highly controlled biomineralisation process similar to the development of inner shell regulated by the mantle^60^. The description of pearl sac collection is explained in the next section. Similarly to other bivalves, the shell of pearl oyster *P. margaritifera* consists of two polymorphs of calcium carbonate: the inner nacreous layer, which is composed of aragonite, and the outer prismatic layer, which is made of calcite^61–63^. Shell formation is a highly controlled process involving multiple matrix proteins^18, 38, 64^. Recently, the number of genes identified as coding for molluscan shell matrix components has increased^65–69^. In order to identify the respective contributions of donor vs. recipient, we sampled tissues of the graft during the graft operation (*i.e*., donor contribution) and tissues of the pearl sac during harvest (*i.e*., recipient and donor contributions) to compare relative gene expression through screening aragonite-related genes (*Pif-177, MSI60, Perline*), calcite-related genes (*Aspein, Shematrin, Prismalin*) and one gene implicated in both layers (*Nacrein*) (Table 2). Two genes were used as housekeeping genes chosen based on their ubiquitous and constitutive expression pattern in *P. margaritifera* tissue: SAGE (SAGES: AGCCTAGTGTGGGGGTTGG/SAGER: ACAGCGATGTACCCATTTCC) (called REF in ref. 22) and GAPDH (GAPDHS: AGGCTTGATGACCACTGTCC/GAPDHR: AGCCATTCCCGTCAACTTC)^70^.

**Table 2:** Set of forward and reverse primers used for the biomineralisation gene expression analysis in *Pinctada margaritifera*.

Primer name	Protein name	Function	GenBank Accession Numbers	Forward primer (5′-3′)	Reverse primer (5′-3′)
PIF	Pif-177	Aragonite formation	HE610401	AGATTGAGGGCATAGCATGG	TGAGGCCGACTTTCTTGG
MSI60	MSI60	Aragonite formation	No accession number but described by B. Marie *et al*.^18^	TCAAGAGCAATGGTGCTAGG	GCAGAGCCCTTCAATAGACC
PERL1	Perline	Aragonite formation	DQ665305	TACCGGCTGTGTTGCTACTG	CACAGGGTGTAATATCTGGAACC
ASP	Aspein	Calcite formation	No accession number but described by B. Marie *et al*.^18^	TGGAGGTGGAGGTATCGTTC	ACACCTGATACCCTGCTTGG
PRISM	Prismalin 14	Calcite formation	HE610393	CCGATACTTCCCTATCTACAATCG	CCTCCATAACCGAAAATTGG
SHEM5	Shematrin	Calcite formation	HE610376	GTCCGAAACCAAATCGTCTG	CTGTGGTGATGGTGACTTCG
CALC1	Nacrein	Aragonite and calcite formation	HQ896199	CTCCATGCACAGACATGACC	GCCAGTAATACGGACCTTGG
SHEM9	Shematrin	Calcite formation	No accession number but described by B. Marie *et al*.^18^	TGGTGGCGTAAGTACAGGTG	GGAAACTAAGGCACGTCCAC

After removing the RNAlater by pipetting and absorption, total cellular RNA was extracted from the individual graft tissue (n = 40) or pearl sac samples (n = 240), using TRIzol® reagent (Life Technologies) according to the manufacturer’s recommendations. RNA was quantified using a NanoDrop ND-1000 spectrophotometer (NanoDrop Technologies, Inc.). Total RNA of each individual was then treated with DNAse I using a DNA-free Kit (Ambion). First, strand cDNA was synthesized from 500 ng total RNA using the Transcriptor First Strand cDNA Synthesis Kit (Roche) and a mix of poly (dT) and random hexamer primers. Real-Time PCR amplifications were carried out on a Roche Light Cycler® 480. The amplification reaction contained 5 μL LC 480 SYBR Green I Mast (Roche), 4 μL cDNA template, and 1 μL of primer (1 µM), in a final volume of 10 μL. Each run included a positive cDNA and a blank control for each primer pair. The run protocol was as follows: initial denaturation at 95 °C for 10 min followed by 40 cycles of denaturation at 95 °C for 30 s, annealing at 60 °C for 30 s and extension at 72 °C for 60 s. Lastly, the amplicon melting temperature curve was analyzed using a melting curve program: 45–95 °C with a heating rate of 0.1 °C s^−1^ and continuous fluorescence measurement. All measurements were made in duplicate and all analyses were based on the Ct values of the PCR products.

Relative gene expression levels were calculated using the delta–delta method, normalized with two reference genes, to compare the relative expression results^71^ as follows:

$${\rm{Relative}}\,{{\rm{expression}}}_{({\rm{target}}{\rm{gene}},{\rm{sample}}{\rm{x}})}={2}^{\hat{\vphantom{a}}-({\rm{\Delta }}{\rm{Ct}}{\rm{sample}},{\rm{sample}}{\rm{x}}-\Delta {\rm{Ct}}{\rm{calibrator}},{\rm{sample}}{\rm{x}})}={2}^{-{\rm{\Delta }}{\rm{\Delta }}{\rm{Ct}}}.$$

Here, the ΔCt calibrator represents the mean of the ΔCt values obtained for the tested gene. The delta threshold cycle (ΔCt) is calculated by the difference in Ct for the target and reference genes. The relative stability of the GAPDH and SAGE combination was confirmed using NormFinder^72^. PCR efficiency (E) was estimated for each primer pair by determining the slopes of standard curves obtained from serial dilution analysis of a cDNA to ensure that E ranged from 90 to 110%. The primers used for amplification are listed in Table 2.

### Recipient and donor influence over kinetic evolution

The experiment was monitored over time to evaluate changes in the recipient/donor influence as the graft became established within the recipient. Two recipient oysters were harvested for each donor after 3, 6, 9, and 12 months of culture. Biometric parameters were measured as described in the section ‘Measurements of shell biometric parameters and pearl growth rate’. Pearls and pearl sac tissue were collected at the same time. Measurement of pearl growth rate and associated quality trait phenotypes were measured as described in sections ‘Measurements of shell biometric parameters and pearl growth rate’ and ‘Cultured pearl quality parameter measurement’. At the time of pearl harvest and in order to minimise the contamination of recipient tissues, we first cut the gonads from the host oysters. We then removed the gonad tissue with a surgical blade until only a thin (<0.5 mm) layer of tissue surrounding the pearls remained. At this point, only the pearl sac and the pearl remain. Next, we made an incision in the pearl sac, removed the pearl, and transferred the pearl sac into a 2.0 ml tube with RNAlater® until RNA extraction^44^ and placed the pearl in a numbered box. While our technique does not completely remove the potential for gonad tissue contamination within pearl sac samples, it does significantly reduce the possibility for gonad contaminations. In addition, the potential for contamination is further reduced because the gonad itself cannot mineralise and thus poses very little risk, as confirmed by Wang *et al*.^73^ who found *MSI60* was not expressed within the gonads of *P. fucata*. A total of 80 pearl sacs were sampled every 3 months, giving a total of 240 pearl sac tissue samples over 9 months (tissues from the last point at 12 months were of too poor a quality to be used in the analyses). Biomineralisation potential for 40 grafts and 217 pearl sacs that contained pearls was screened via gene expression as described in section ‘Biomineralisation gene expression analysis’ (Fig. 6).

### Statistical analysis

The normality of the data distribution and homogeneity of variance were tested for pearl size, donor, recipient oyster biometric parameters and relative gene expression data using the Shapiro‒Wilk test and Bartlett’s test. When necessary, transformations were used to adjust data to this distribution (logarithm or square roots). Group donor size (QL vs. TL) and time harvest were treated as fixed variables for statistical analysis, with donor treated as a random variable unless otherwise stated. A linear mixed effects model was used to assess the effect of the fixed and random variables on nacre weight, thickness and relative gene expression, and a generalized linear mixed effects model (family = binomial) was used for the presence or absence of circles and luster, both using packages lme4 and nlme^74^ with the R© software. Qualitative classes based on cultured pearl surface defects, grade and darkness level were re-encoded into ordered categorical response variable. Scores from 0 to 4 were attributed to the different classes from the least to the most valuable. Analyses of ordered categorical response variables (pearl darkness, surface defects and grade) were analyzed using cumulative link mixed models implemented in the ordinal package^75^ with the R© software. The significance of fixed variables was tested by comparing the fit of an all-inclusive model with that of a reduced model for each variable using a fisher test or chi-squared test. For the cultured pearl “color categories” and shape categories, donor and time of harvest effect were compared using χ^2^ tests.

Spearman’s correlation coefficient was used to evaluate the correlations between nacre weight and thickness, and the four biometric measures of the recipient and donor oysters, and gene expression. We have adjusted the p-value with Bonferroni correction (level of significance = α/c, in which α = 0.05, and c = number of comparisons performed) to compare pairwise correlation in multiple comparison. The gene expression values of each graft donor and pearl sac were associated with the corresponding nacre weight and thickness of each pearl. The statistics and the visualization for the correlation matrix (Fig. 3) were done with the R© software Hmisc and Corrplot package using the spearman method^76, 77^.

In all cases, the differences were considered statistically significant when *p* values were lower than 0.05. Statistical analyses were performed using XLSTAT (version 2009.4.02) and R© software (version 3.2.1).

## Electronic supplementary material

Supplementary Table S1
