# Supplementary material for: Donor and recipient contribution to phenotypic traits and the expression of biomineralisation genes in the pearl oyster model Pinctada margaritifera
Source: Sci Rep. 2017 Jun 2;7:2696. doi: 10.1038/s41598-017-02457-x (PMC5457395; doi:10.1038/s41598-017-02457-x)
Supplement: Supplementary file 1 — Supplementary Table S1 [file 41598_2017_2457_MOESM1_ESM.pdf]

Donor and recipient contribution to phenotypic traits and the expression of biomineralisation genes in the pearl oyster model *Pinctada margaritifera*

Carole BLAY[1,2,3], Serge PLANES[2, 3] and Chin-Long KY[1, 3*]

Supplementary Table S1

Summary of statistical data between pearl quality traits and the relative expression of 8 selected genes implicated in the biomineralization processes in *P. margaritifera* mantle graft tissue and all pearl sacs. Statistical differences are indicated in bold after Bonferroni correction with the value of significance level ($p < 0.0004$) or "ns" for non significant values ($p > 0.0004$).

		Shape	Circle	Surface defect	Lustre	Grade	Darkness	Colour
Graft	PIF	ns	ns	ns	ns	ns	ns	ns
	MSI60	ns	ns	ns	ns	ns	ns	ns
	PERL1	ns	ns	ns	ns	ns	ns	0.002^{ns}
	ASP	ns	ns	ns	ns	ns	ns	ns
	PRISM	ns	ns	ns	ns	ns	ns	ns
	SHEM5	ns	ns	ns	ns	ns	ns	ns
	CALC1	ns	ns	ns	ns	ns	ns	ns
	SHEM9	ns	ns	ns	ns	ns	ns	ns
Pearl sac	PIF	ns	ns	**<0.0001**	**<0.0001**	**<0.0001**	0.019^{ns}	**0.0002**
	MSI60	ns	ns	**<0.0001**	0.001^{ns}	0.03^{ns}	ns	**<0.0001**
	PERL1	ns	ns	0.001^{ns}	0.001^{ns}	ns	ns	**<0.0001**
	ASP	0.013^{ns}	ns	**<0.0001**	**<0.0001**	**<0.0001**	0.002^{ns}	**<0.0001**
	PRISM	0.037^{ns}	ns	0.002^{ns}	**<0.0001**	**<0.0001**	0.007^{ns}	0.008^{ns}
	SHEM5	ns	0.043^{ns}	0.026^{ns}	**<0.0001**	0.01^{ns}	0.037^{ns}	ns
	CALC1	ns	ns	ns	0.014^{ns}	ns	0.002^{ns}	**0.0003**
	SHEM9	0.01^{ns}	ns	**0.0002**	**<0.0001**	**<0.0001**	0.013^{ns}	0.001^{ns}
